# Brain Representation in Conscious and Unconscious Vision

**DOI:** 10.5334/joc.443

**Published:** 2025-04-28

**Authors:** Ning Mei, David Soto

**Affiliations:** 1 School of Psychology, Shenzhen University, No. 3688, Nanhai Avenue, Shenzhen 518060, China; 2 Basque Center on Cognition, Brain and Language, San Sebastian, Spain; 3 Ikerbasque, Basque Foundation for Science, Bilbao, Spain

**Keywords:** Consciousness, fMRI, Visual perception

## Abstract

The development of robust frameworks to understand how the human brain represents conscious and unconscious perceptual contents is paramount to make progress in the neuroscience of consciousness. Recent functional MRI studies using multi-voxel pattern classification analyses showed that unconscious contents could be decoded from brain activity patterns. However, decoding does not imply a full understanding of neural representations. Here we re-analysed data from a high-precision fMRI study coupled with representational similarity analysis based on convolutional neural network models to provide a detailed information-based approach to neural representations of both unconscious and conscious perceptual content. The results showed that computer vision model representations strongly predicted brain responses in ventral visual cortex and in fronto-parietal regions to both conscious and unconscious contents. Moreover, this pattern of results generalised when the models were trained and tested with different participants. Remarkably, these observations results held even when the analysis was restricted to observers that showed null perceptual sensitivity. In light of the highly distributed brain representation of unconscious information, we suggest that the functional role of fronto-parietal cortex in conscious perception is unlikely to be related to the broadcasting of information, as proposed by the global neuronal workspace theory, and may instead relate to the generation of meta-representations as proposed by higher-order theories.

## Introduction

The neuroscience of visual consciousness seeks to elucidate the neural underpinnings of subjective experience. To achieve this goal, it is paramount to develop robust frameworks for distinguishing unconscious and conscious information processing. The present study embraces an information-based approach, integrating machine learning classification procedures and computational modeling techniques with functional Magnetic Resonance Imaging (fMRI) data to understand the properties of unconscious representations in the human brain.

The extent to which conscious and unconscious visual contents are processed in the higher-level stages of the ventral visual stream and associated parieto-prefrontal areas remains the subject of ongoing debate (Boly et al., 2017; Brown et al., 2019; Dehaene, 2014; Lau, 2022). Previous neuroimaging work has shown that object categories of visible stimuli are encoded in the ventral-temporal cortex (Kriegeskorte, 2011; Naselaris et al., 2011) and perceptual and mnemonic contents can also be decoded in fronto-parietal cortex (Christophel et al., 2012; Ester et al., 2015; Kapoor et al., 2020; Panagiotaropoulos et al., 2012). It has been argued that unconscious contents are temporarily and locally encoded in the visual cortex (Lamme, 2020), while visual consciousness may be associated with activity in large-scale association networks, involving fronto-parietal cortex (cf. the global neuronal workspace model, (Dehaene & Changeux, 2011). However, recent research suggests that unconscious information processing might support higher-order cognition (Berkovitch & Dehaene, 2019; Chong et al., 2014; Güldener et al., 2022; Lau & Passingham, 2007; Rosenthal et al., 2016; Soto et al., 2011; Trübutschek et al., 2017; Van Gaal & Lamme, 2012; Van Gaal et al., 2014; Wuethrich et al., 2018). Nevertheless, the purported influence of unconsciously processed information on behavioural responses has proven challenging to replicate (Stein et al., 2020a). Unconscious processing research has been heavily criticised on methodological grounds related to the low number of trials used in objective awareness tests to exclude conscious awareness, and the use of subjective measures of awareness that are known to be influenced by criterion biases (Newell & Shanks, 2014; Shanks et al., 2021).

One reason for the ongoing controversy around the scope of unconscious information processing is the absence of robust frameworks to effectively isolate the influence of unconscious contents at behavioural and neural levels (Soto et al., 2019; Stein et al., 2020b). Research on unconscious processing is heavily constrained by the low signal to noise ratio of the unconscious stimulus, which is typically presented very briefly and/or strongly masked. If the unconscious item is poorly represented in the brain, it is likely that unconscious effects on behaviour are only weak or difficult to detect (Lau, 2022). However, the absence of observable effects on behavior does not refute the existence of unconscious processing. The brain-based framework to unconscious processing (Soto et al., 2019) suggests that information-based analysis of brain activity patterns can provide insights into the representations of unconsciously processed stimuli, even with null effects on behavioral measures.

A recent fMRI study employing a high-precision (N = 7), within-participant approach to investigate the neural representation of unconscious contents (Mei et al., 2022b) showed that unconscious contents, even those associated with null sensitivity, can be reliably decoded from multi-voxel patterns that are highly distributed along the ventral visual pathway and also involving parieto-frontal substrates. It should be noted, however, that the capacity to decode specific information does not equate to a comprehensive understanding of neural representations (Kriegeskorte & Douglas, 2018). Hence, beyond linear decodability, pattern classification approaches fall short to delineate how specific representations of perceptual content is encoded in the brain. In contrast, encoding models and representational similarity analysis (RSA Kriegeskorte et al., 2006) offer more refined approaches for pinpointing the neural representation of perceptual input. In the current study, we re-analysed fMRI data from our previous study (Mei et al., 2022b) in which participants underwent six fMRI sessions across six days while performing a discrimination task for animate and inanimate images presented very briefly and masked. Model-based RSA (Kriegeskorte, 2011; Kriegeskorte et al., 2008) was used to provide a fine-grained information-based approach to the neural representation of (un)conscious and conscious contents. RSA can be used to quantify the geometry of multi-voxel patterns in a given brain region relative to a computational model (Diedrichsen & Kriegeskorte, 2017; Kriegeskorte et al., 2008). In order to model the brain responses to the visual images, both seen and unseen, we assessed the correspondence of the multi-voxel response patterns elicited by the images and the representation of the images given by convolutional neural network models (CNNs), which have been successfully used to explain brain activity in object recognition tasks (Güçlü & van Gerven, 2015; Khaligh-Razavi & Kriegeskorte, 2014; Kriegeskorte, 2009; Lindsay, 2021; Schrimpf et al., 2020a; Seeliger et al., 2018). Further, by applying a whole-brain searchlight approach (Kriegeskorte et al., 2006), we provided a broader and more detailed examination of shared neural representations. Following the decoding results from Mei et al. (2022b), here we predicted that the brain representation of unconscious contents, indexed by the similarity between a computational vision model and the brain activity patterns, to be also broadly distributed and involve fronto-parietal regions. We also predicted that model-based representations of conscious perceptual content would generalise to predict the representation of unconscious perceptual content. Furthermore, the present study also performed cross-validation of the models across the different participants (i.e. training the model in a given participant and testing the models in a different participant), going beyond our previous within-subject approach (Mei et al., 2022b). This approach also allowed us to investigate whether there is a common neural representation of conscious and unconscious knowledge shared across participants.

## Methods

### Experimental paradigm

Seven participants were presented with 96 images, equally divided into animate and inanimate categories, which could be further grouped into 10 subcategories (e.g., animals, vehicles) and 2 overarching categories (animate vs. inanimate). The experiment was conducted over six days, with each day consisting of nine blocks of 32 trials, totaling 288 trials per day and 1728 trials per participant.

**Figure 1:**
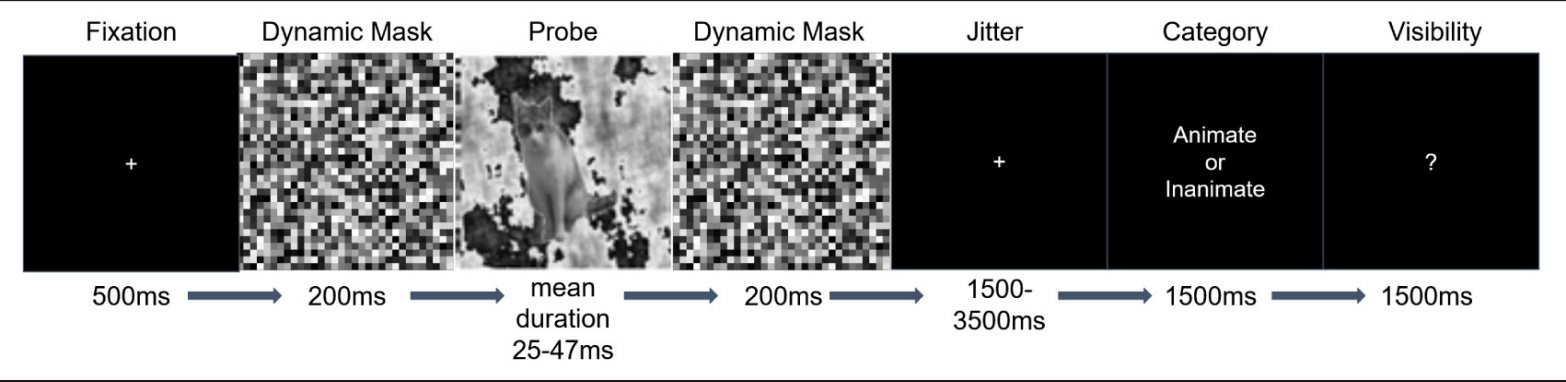
**Experimental paradigm**. Example of the sequence of events within an experimental trial. Participants were asked to discriminate the category of the masked image (animate v.s. inanimate) and then rate their visual awareness on a trial by trial basis.

Participants were asked to discriminate whether the target images were animate or inanimate (Moreno-Martínez & Montoro, 2012). The images were grayscale, varied in orientation, and augmented using TensorFlow-Keras. A random-phase noise background, generated from the original images, was added for masking. The experiment, programmed with Psychopy v1.83.04 (Peirce, 2007), was displayed on a 100 Hz monitor. Each trial began with a 500 ms fixation point, followed by a 500 ms blank screen, 20 frames of Gaussian noise, a briefly presented probe image, another 20 frames of Gaussian noise, and a jittered blank period ranging from 1500 to 3500 ms before the perceptual decisions were made. A further inter-trial jitter of 6000–8000 ms was implemented. The asynchrony between probe images across successive trials therefore ranged between 11.5 and 15.5 seconds to minimize carryover effects. Participants discriminated whether the probe image presented on each trial was animate or inanimate, and also rated their visual awareness using the following categories: (i) no experience/just guessing, (ii) brief glimpse, and (iii) clear experience with a confident response. Each of the perceptual identification and awareness response periods had a 1500 ms deadline. The duration of the images was based on an adaptive staircase that was running throughout the experiment, designed to obtain a high proportion of unconscious trials. On average, target duration was (i) 25 ms on trials rated as unaware, (ii) 38 ms on glimpse trials, and (iii) 47 ms on aware trials. Figure 1 illustrates an example of a trial in the behavioral task. Additional details can be found in Mei et al. (2022b).

### Encoding-based RSA pipeline

Standard RSA characterizes the structure of cognitive or perceptual representations within a feature space in terms of distances between response vectors associated with different aspects of the task or the experimental stimuli (Kriegeskorte et al., 2008). This is measured by distance-based measurements such as Euclidean distance (Kriegeskorte et al., 2006) between neural activity patterns for each pair of experimental conditions or for each pair of representations given by a computational model. Konkle and Alvarez (2022) proposed an encoding-based RSA pipeline in which encoding models are added on top of the standard RSA pipeline. The encoding models help to better contextualize how the representations given by computational models (i.e. convolutional neural networks, such as ResNet50 (He et al., 2016)) relate to the brain representations. In order to allow for robust statistical inference in our highly-sampled within-subject design, the encoding model of the RSA pipeline was fitted and tested across pairs of participants. Standard RSA pipelines are not designed to perform within-subject statistical inference. Descriptive results from exploratory RSA pipelines assessed in a within-subject manner are reported in Mei et al. (2022a). Here we present an improved encoding-based RSA framework using a cross-participant cross-validation approach. This approach produces independent RSA maps across participants that could be fed to statistical permutation tests.

#### Extraction of the representations of the images from computer vision models

ResNet50 has been consistently ranked one of the best models in predicting brain responses during object recognition (Schrimpf et al., 2018, 2020a). We elected to use this model in the current study based on these factors. First, ResNet50 has proven excellent performance and reliability, demonstrated by its high ranking in predicting brain activity (Nonaka et al., 2021), thereby ensuring that it can effectively model brain responses to visual stimuli. Second, the deep hierarchical structure and residual connections of ResNet50 allow it to capture both low-level and high-level features (Schrimpf et al., 2018, 2020b), making it suitable for the complexity of brain data. Third, ResNet50 is pre-trained on large datasets like ImageNet (Deng et al., 2009), which provides a strong foundation for transfer learning (Yosinski et al., 2014), allowing us to fine-tune the model on our specific dataset with fewer resources. Fourth, ResNet50’s architecture supports feature visualization and interpretability, aiding in understanding and validating the model’s predictions (Nonaka et al., 2021). Furthermore, the wide adoption and extensive documentation of ResNet50 offer a wealth of resources and community support, facilitating its integration and optimization. Finally, ResNet50 has a balance between performance and computational efficiency, making it a practical and scalable choice for our research. The “50” in ResNet50 denotes the approximate depth of the network (i.e. around 50 layers). ResNet50’s architecture is characterized by its repetitive use of residual blocks with skip connections, enabling the training of very deep networks without degradation problems, and achieving high performance in image recognition tasks.

First, we fine-tuned the pre-trained ResNet50 architecture (He et al., 2016), which was initially trained on ImageNet (Deng et al., 2009), with the Caltech101 dataset (Fei-Fei et al., 2004) to reduce the dimensionality of hidden representations to 300. During fine-tuning, the pretrained ResNet50 (He et al., 2016) was stripped of the original fully-connected layer while weights and biases of the convolutional layers were frozen and not updated further (Yosinski et al., 2014). An adaptive pooling (McFee et al., 2018) operation was applied to the last convolutional layer so that its output became a one-dimensional vector, and a new fully-connected layer took the weighted sum of the previous outputs (i.e. the ‘hidden layer’). The outputs of the hidden layer were passed to a Scaled Exponential Linear Unit (SELU) activation function (Klambauer et al., 2017).

A new fully-connected layer, namely, the classification layer, took the outputs processed by the activation function of the hidden layer to compose the classification layer. The model was trained using 96 unique categories of the Caltech101 images (BACKGROUND_Google, Faces, Faces_easy, stop_sign, and yin_yang were excluded). The convolutional layers were frozen during the training, while the weights of the newly added two layers were modified. The loss function was binary cross entropy and the optimizer was Stochastic Gradient Descent. The data was split into train and validation partitions, and the training was terminated if the performance on the validation data did not improve for five consecutive epochs.

**Figure 2:**
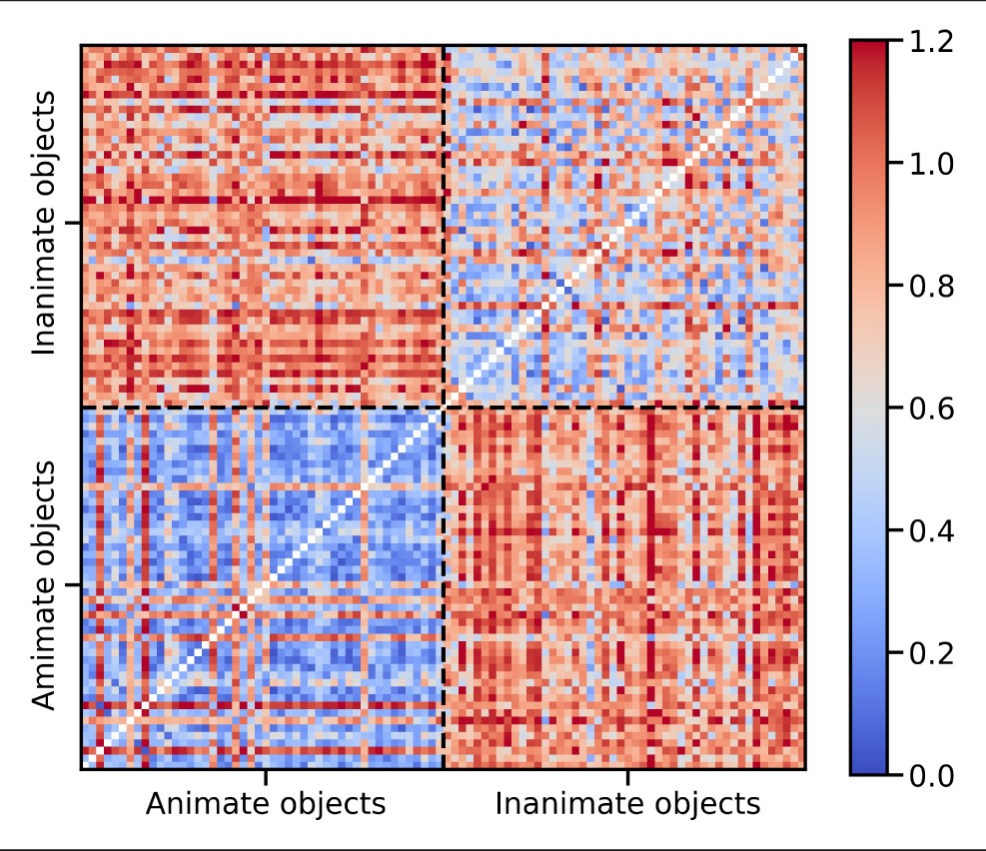
Representational dissimilarity matrix of the hidden representations of the ResNet50 model fine-tuned by Caltech101 dataset. The images were resized to 128 × 128 × 3 and then passed through the model to obtain the feature representations. The RDM was computed by 1 – Pearson correlations of the feature representations. The first 48 items were animate and the last 48 items were inanimate.

We then fed the trained FCNNs with exactly the same images used in our experiment but without the noise background, in order to extract the hidden representations of the images. We then averaged the hidden representations of the trials belonging to the same item (i.e cat) and computed the model representational dissimilarity matrices (RDMs) for the ResNet50 (Figure 2 depict the RDM for ResNet50 hidden representations), where they appear clear clusters for the animate and inanimate image categories. The similarities were more consistent among animate images (lower left quadrant) compared to the inanimate images (upper right quadrant).

Regarding the RSA metric we elected to use 1 – Pearson correlation since this is most common in the literature and it is computational efficient (Diedrichsen & Kriegeskorte, 2017; Nili et al., 2014). The other popular choices include Euclidean distance and its second-momentum derivation Mahanalobis distance (Diedrichsen & Kriegeskorte, 2017), but as noted before, the Pearson correlation is the standard approach in fMRI studies to compute the RDMs. Following this, we used Spearman correlation to compare the RDMs (i.e. based on the observed and predicted responses), which is also a standard approach (Diedrichsen & Kriegeskorte, 2017).

#### Cross-validation of the encoding-based RSA across participants

A standard RSA pipeline involves computing a model representational dissimilary matrix (RDM) and a corresponding brain RDM, and then compute the similarity (i.e. Pearson correlation) between the model and the brain RDMs (Kriegeskorte, 2015; Kriegeskorte & Douglas, 2019; Kriegeskorte et al., 2008). In our previous preprint (Mei et al., 2022a), we used a standard RSA approach in an exploratory manner, performed within each subject only for descriptive purposes. In the current study, we conducted a cross-validated encoding-based RSA that was trained and tested between pairs of subjects to reveal robust and reliable patterns across subjects.

**Figure 3:**
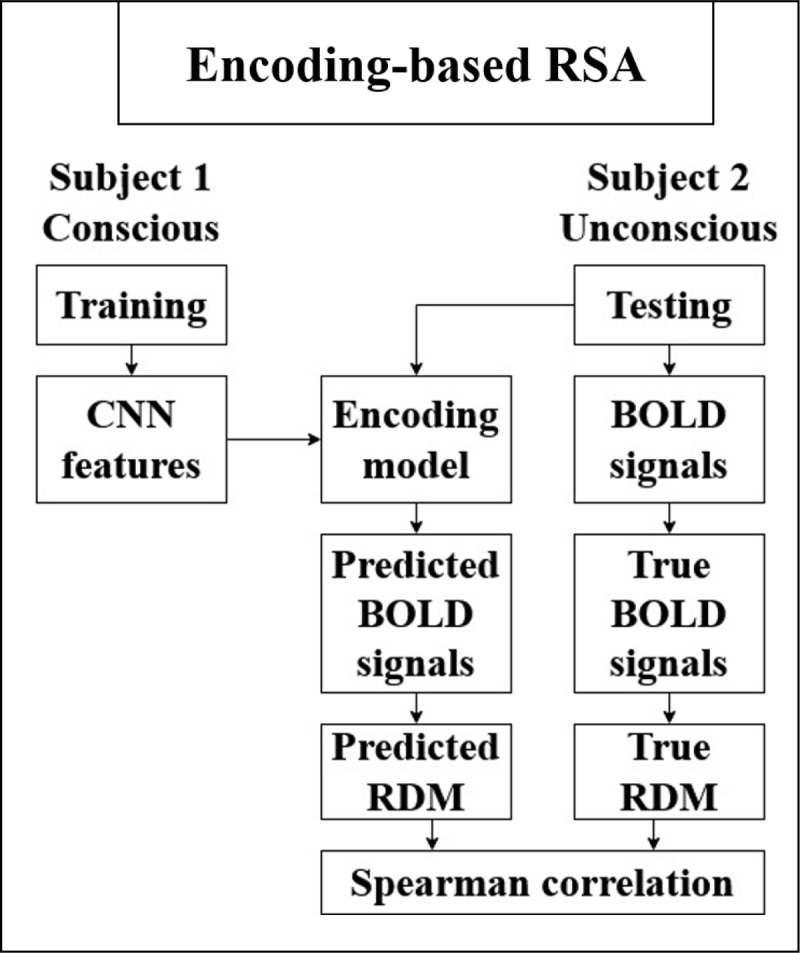
**Illustration of the pipeline used for encoding-based RSA across subjects and awareness states (i.e. from conscious to unconscious) or within the conscious or within the unconscious trials (not shown in the Figure)**. First, an encoding mode is trained to predict voxelwise responses based on the hidden layer representation of a convolutional neural network (CNN). The model is trained on the conscious trials of one participant and tested on the unconscious trials of a different participant in a standardized brain space. The predicted BOLD responses are then used to compute a predicted RDM across item pairs for a given searchlight sphere. The lower fields of computing the correlation between the predicted and true RDMs constitute the RSA.

The encoding-based RSA pipeline starts by fitting an encoding model to predict voxel-wise wise responses (Konkle & Alvarez, 2022). Then, the RSA is computed between the predicted brain responses and the actual brain responses using a whole-brain searchlight algorithm. The encoding-based RSA was further cross-validated across participants. For each cross-validation fold, trials from a specific awareness condition (e.g., conscious) belonging to one participant were utilized to train an encoding model. This encoding model used features extracted from a computer vision model to predict voxel-wise BOLD responses. Following this, the encoding model was applied to trials from the same awareness condition (e.g., unconscious) but belonging to a different participant. The observed and predicted BOLD responses were summarized for each of the visual items leading to a 96 × 96 RDMs based on the observed and the predicted neural responses. The lower triangular parts of these two RDMs were correlated using Spearman. The entire analysis process was conducted following the normalization of each individual brain data to the standard Montreal Neurological Institute (MNI, Mazziotta et al., 2001; Mazziotta et al., 1995) space utilizing a searchlight sphere with a radius of 6 mm (see below). The Spearman correlation coefficients were assigned to the center of the sphere (Figure 3). The normalization ensured that the input size for fitting the encoding model in a given subject and for testing (predicting) in different subjects is the same.

The single-session fMRI data were transformed to standard space using a two-step registration process with FLIRT (FMRIB’s Linear Image Registration Tool) in FMRIB Software Library (FSL, Jenkinson et al., 2012). First, each subject’s fMRI data were aligned to their corresponding high-resolution structural MRI scan using Boundary-Based Registration (BBR) with 7 degrees of freedom (DOF). Next, the high-resolution structural MRI was registered to the MNI 152 space (average brain atlas from the McConnell Brain Imaging Center, Montreal Neurological Institute average), using a 12 DOF linear transformation. The resulting transformation matrices were used to normalize the individual fMRI data to standard space.

The searchlight analyses were then conducted in standard space. The searchlight algorithm used a sphere of 3 voxels moving across the whole brain (Kriegeskorte & Bandettini, 2007; Kriegeskorte et al., 2006) in which the RSA or decoding analysis was performed (Kriegeskorte et al., 2006).

Three distinct cross-participant validation scenarios for the encoding-based RSA were employed: (1) within-conscious-state cross-validation, (2) within-unconscious-state cross-validation, and (3) conscious-to-unconscious generalization.

In summary, in each of the analyzes, we first generated decoding or RSA maps from all possible pairwise combinations of subjects (i.e., training the model in subject X and testing the model in subject Y. Since there are 7 participants, this results in 42 independent maps resulting from training and testing the model across different observers. These maps are concatenated and fed into a nonparametric permutation test (i.e. using Randomise in FSL) to assess the statistical significance, using Threshold Free Cluster Enhancement (TFCE). TFCE is a statistical method used to enhance the detection of fMRI signals without setting arbitrary cluster-forming thresholds (Smith & Nichols, 2009). TFCE integrates spatial neighborhood information to boost sensitivity to true signals compared to voxel-wise or traditional cluster-based thresholding. Permutation testing is performed to establish the significance of the TFCE statistics (Smith & Nichols, 2009).

### Decoding pipeline

We performed a searchlight analysis to pinpoint the brain regions whose activity patterns discriminated the animate versus inanimate dimension of the image. We used a similar between-participant cross-validation method as described in the encoding-based RSA methods. Each participant’s fMRI dataset was normalized to the standard MNI space prior to conducting the analysis (Collins et al., 1994).

The decoding analyses employed the searchlight algorithm using the voxels within each sphere as features for the decoding analysis. The decoding analyses were conducted in pairs of subjects. Trials from one subject were used for training a decoder to classify the category of the items (animate vs inanimate), while trials from another subject were used as the testing set. The trained decoder predicted the labels of the testing trials. The decoding model comprised a scalar for feature normalization and an L1-regularized linear support vector machine (SVM) for classification. During classifier training, the scalar derived means and standard deviations were used to standardize the features. These parameters were locked during the test. We used L1-regularization to facilitate feature selection by assigning zero weights to sparsity—irrelevant features (Vidaurre et al., 2013). The predicted labels and the true labels were used to compute the decoding performance. We chose the area under the Receiver Operating Characteristic Curve (ROC AUC), an unbiased metric for binary classification tasks (King et al., 2016).The ROC AUC score was then assigned to the center of the searchlight sphere. The searchlight was centered at each voxel in turn to produce a decoding brain map.

The decoding analysis also included cross-validation procedures across the different awareness states (i) within the conscious trials, (ii) within the unconscious trials, and (iii) from conscious to unconscious trials. For each cross-validation procedure, 42 brain maps were produced resulting from the combination of subject pairs used for training and testing the decoder. A permutation t-test using Randomise from FSL with TFCE was used to assess the statistical significance of the voxels in which the decoding scores were greater than 0.5.

## Results

The signal detection theoretic analyses of the behavioural data can be found in Mei et al. (2022b). In short, four of the participants showed null perceptual sensitivity of the stimulus category on the trials rated as unaware, while the remaining three participants showed ’blindsight’ like behaviour (i.e. above chance discrimination performance on trials reported as unaware). In the following presentation of the results we use the label ‘unconscious’ to refer to the trials in which the participants reported no experience of the visual stimulus and ‘conscious’ to refer to the trials in which the subjects reported clear experience.

### Encoding-based RSA

As noted in the introduction, multivariate pattern classification analyses (i.e. decoding) may shed light on the informational content that is present within brain activity patterns. but fails to provide a fine-grained, interpretable account of neural representations. Encoding models and Representational Similarity Analysis (RSA) provide more sophisticated methods for exploring how the brain represents information. Building on the work of Konkle and Alvarez (2022), we incorporated a cross-participant validation approach within an encoding-based RSA framework. We trained the encoding model with the trials of a given awareness condition (i.e., conscious) from a given participant. Then, the model was used to predict the BOLD signals of a different participant in a given awareness condition (i.e., unconscious trials). The predicted BOLD signals, in conjunction with the actual BOLD responses from the test participant, served as inputs for conducting a whole-brain searchlight RSA analysis (see Methods).

**Figure 4:**
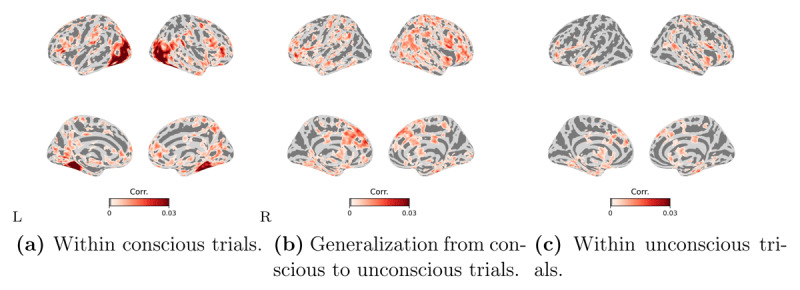
**Searchlight encoding-based RSA results in different cross-validation procedures**. **(a)** within the conscious trials, **(b)** generalization from conscious to unconscious trials, and **(c)** within the unconscious trials. The Spearman’s rank correlation is computed between the predicted RDM based on the encoding model and the true RDM based on the BOLD responses of each moving searchlight sphere. Correlation coefficients were assigned to the center of the moving searchlight sphere. We show significant clusters that have been whole-brain corrected using TFCE (*p* < 0.05). The color bars show the range of correlation coefficients between 0 and 0.03. The left/right images of each panel display the left/right hemisphere. Corr. on the colorbar stands for correlation coefficient.

In the conscious trials, there was a highly distributed set of regions involving the ventral visual pathway and fronto-parietal areas in which BOLD responses significantly correlated with the computer vision model representations (Figure 4a). The generalization from conscious to unconscious trials showed a similar distributed profile (Figure 4b), which, remarkably, was also confirmed by the analysis restricted to the unconscious trials (Figure 4c).

### Decoding results

Building on the encoding-based RSA results showing the highly distributed representation of unconscious content, we employed a decoding approach to validate the results, and in particular, to further test the possibility that a common neural architecture supporting the representation of unconscious (and conscious) contents is shared across individuals. Accordingly, similar to the encoding-based RSA analyses, here we used a cross-validation scheme in which the decoder was trained and tested across different pairs of participant.

**Figure 5:**
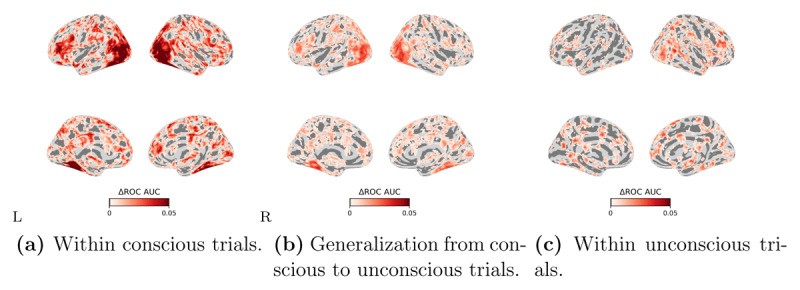
**Searchlight decoding results in different cross-validation procedures**. **(a)** within the conscious trials, **(b)** generalization from conscious to unconscious trials, and **(c)** within the unconscious trials. Decoding was conducted using a searchlight algorithm. ΔROC AUC on the colorbar represent the difference between the empirical ROC AUC (Receiver Operating Characteristic Area Under the Curve) and the theoretical chance-level score (0.5). The red color scale (0 to 0.05) corresponds to absolute differences from the chance level. A score of 0.05 indicates a 5% improvement over chance (i.e., ROC AUC = 0.55). the upper limit of the color bar is 0.05 (ROC AUC = 0.55). This cap was set to emphasize the range of biologically plausible effects, though individual voxels within significant clusters may exceed this value. The lower limit is 0 (ROC AUC = 0.5). ΔROC AUC scores were assigned to the center of the moving searchlight sphere. We show significant clusters that have been whole-brain corrected using TFCE (*p* < 0.05). The left/right images of each panel display the left/right hemisphere.

The searchlight decoding analysis showed that the stimulus category could be decoded from activity patterns in the ventral visual pathway, parietal, and prefrontal cortex. All these areas contained activity patterns that predicted the stimulus category and that generalized awareness states and across participants (Figure 5a). The generalization from conscious to unconscious trials showed a similar distributed pattern of brain representation, including the ventral visual pathway, parietal, and prefrontal cortex (Figure 5b), which also held when the analysis was performed on the unconscious trials (Figure 5c).

### Encoding-based RSA and decoding analysis for participants who showed null perceptual sensitivity

We then performed the analyses on the four participants that showed null perceptual sensitivity (i.e. no significantly greater than zero in stratified permutation tests; see Mei et al. (2022b)). Accordingly, we excluded the three participants showing above chance perceptual sensitivity on trials in which they reported to be unaware of the target stimulus. In this way, we could rule out the potential influence of the criterion biases in reporting the absence of awareness and hence the possibility that above chance perceptual sensitivity may reflect some degree of “conscious” influence that could also conflate the observed brain activity patterns.

**Figure 6:**
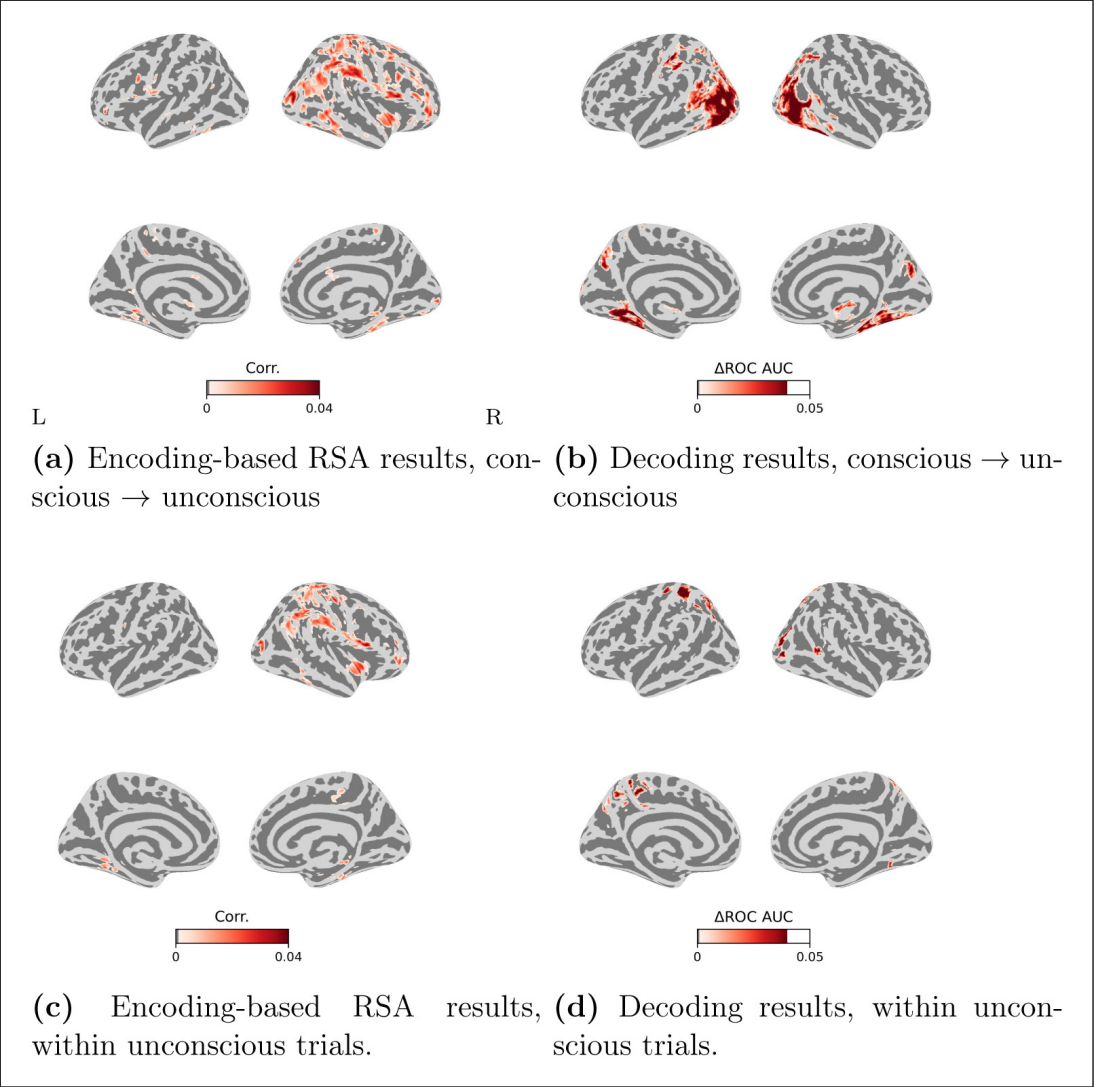
**Results from the encoding-based searchlight RSA and decoding on the observers that showed null perceptual sensitivity**. **(a)** Encoding-based RSA results, generalization from conscious trials to unconscious trials; **(b)** Decoding results, generalization from conscious trials to unconscious trials; **(c)** encoding-based RSA results within unconscious trials; and **(d)** decoding results within unconscious trials. ΔROC AUC on the colorbar represent the difference between the empirical ROC AUC (Receiver Operating Characteristic Area Under the Curve) and the theoretical chance-level score (0.5). Corr. on the colorbar stands for Spearman Rank correlation coefficient. The red regions show clusters where the Correlation coefficients or ROC AUC scores are significantly higher than chance level. Correlation coefficients or ROC AUC scores were assigned to the center of the moving searchlight sphere. We show significant clusters that have been whole-brain corrected using TFCE (*p* < 0.05). The color bars show the range of correlation coefficients or ROC AUC scores between 0 and 0.04. The left/right images of each panel display the left/right hemisphere.

The results are presented in (Figure 6). Overall, the pattern of results is similar to the previous analysis with the full sample of participants. The encoding-based RSA results showed again a distributed set of regions involving the ventral visual pathway and fronto-parietal areas in which BOLD responses significantly correlated with the representations of the items given by the CNN model, both when the encoding models was fitted on conscious trials and tested on the unconscious trials (Figure 6a), and also when the analysis was restricted to the unconscious trials (Figure 6b). The decoding results also showed that the unconscious content could be decoded from clusters in visual and parietal areas, both when the decoder was tested on the conscious trials and tested on the unconscious trials (Figure 6c) and also when the decoding analysis was restricted to the unconscious trials (Figure 6d). We also note the absence of significant decoding performance based on prefrontal activity patterns. However, it should be noted that by reducing the sample size to four participants, the number of folds for the cross-validation was also smaller, thereby reducing the chances of detecting significant results.

## Discussion

Developing robust paradigms for assessing the brain representation of unconscious and conscious contents is critical to make progress in the neuroscience of consciousness at both theoretical and empirical levels. Here, we employed an information-based approach, using fMRI in conjunction with model-based RSA and decoding pipelines, to investigate the brain representation of visual objects across different awareness states.

The encoding-based RSA searchlight showed that computer vision model representations strongly correlated with neural responses during object recognition in ventral visual cortex. This result aligns with prior work demonstrating that CNNs are a good computational model of the ventral visual pathway (Güçlü & van Gerven, 2015; Khaligh-Razavi & Kriegeskorte, 2014; Kriegeskorte, 2015; Yamins et al., 2013; Yamins & DiCarlo, 2016). The encoding-based RSA results revealed a highly distributed set of brain regions, including fronto-parietal cortex, involved in the representation of both conscious and unconscious perceptual input. It may be argued that a representational account of brain function also requires that the encoded information guides behavioral performance (Kriegeskorte & Diedrichsen, 2019; Shea, 2018). However, whether or not strongly masked (unconscious) information influences behavior may depend of different factors (e.g. related to brain state) that remain to be determined (Soto et al., 2019). Our pattern of results indicate that unconscious perceptual contents are indeed represented in brain activity, which may have behavioural consequences under certain experimental conditions.

In keeping with the encoding-based RSA, the decoding results showed that, in both conscious and unconscious trials, the stimulus category could be decoded in a highly distributed set of brain regions involving the ventral visual pathway and fronto-parietal substrates. Moreover, the decoding analysis confirmed the significant generalization of a model trained on the conscious trials and tested on the unconscious trials that was also observed in the RSA. This observation is in line with our previous fMRI study using a within-subject analytical approach based on regions of interest (Mei et al., 2022b), in which we showed that unconscious contents are decodable in fusiform, parietal and prefrontal cortex in most of the participants, with significant generalization from conscious to unconscious trials.

These observations are in contrast to prior reports that neural markers of unconscious information processing are restricted to visual cortical regions (Dehaene et al., 2001; Jiang et al., 2007; Ludwig & Hesselmann, 2015; Moutoussis & Zeki, 2002; Stein et al., 2021). However, a few fMRI studies have previously reported prefrontal involvement during unconscious processing in syntactic and semantic tasks (Axelrod et al., 2014; Sheikh et al., 2019), visual short-term memory (Bergström & Eriksson, 2017; Dutta et al., 2014) and cognitive control (Güldener et al., 2022; Lau & Passingham, 2007; Van Gaal et al., 2008). The increased number of trials per participant in the study by Mei et al. (2022) may well explain the higher sensitivity to decode the content of unconsciously processed information across the brain.

Notably, the decoding and the encoding-based RSA was fitted using brain responses from a given participant and then tested on a different participant. The results from both approaches reveal a notable degree of similarity across participants in the neural representations of conscious and unconscious content. A recent fMRI study using an encoding model with visible images has shown a higher inter-individual variation in the representations in the prefrontal cortex (Lin & Lau, 2024) relative to the ventral visual cortex, which appears more stable across participants. The present results indicate that despite the potential inter-subject variability in the prefrontal representation of visual content, there appears to be a common blueprint in the brain that supports a common, shared representations across participants and states of visual awareness. Methodological considerations should also be noted. As described in our methods, while we selected 1-Pearson correlation distance for its interpretability when comparing representations across different metrics (e.g., Euclidean vs. Riemannian distances; Walther et al., 2016), we acknowledge three key limitations of this approach. First, Pearson correlation captures only linear relationships between representations, potentially overlooking nonlinear structures in neural coding (Williams et al., 2021). Second, its sensitivity to outliers and assumption of homoscedasticity may distort similarity estimates in noisy fMRI data. Third, as a rotation-sensitive metric, it cannot account for geometrically equivalent representations under orthogonal transformations (Shahbazi et al., 2021). Future studies could extend this framework by incorporating Riemannian manifold distances or generalized shape metrics to better characterize hierarchical representational geometries across cortical hierarchies.

The involvement of fronto-parietal cortex in the representation of unconscious content, along with the significant generalization between conscious and unconscious representations, suggests that the functional role of fronto-parietal cortex in conscious perception is unlikely related to the broadcasting of information, as proposed by the global neuronal workspace model (Dehaene et al., 1998). According to the global neuronal workspace model (GNWT), conscious awareness arises from sustained activity within large-scale association networks, particularly involving the fronto-parietal cortex. This activity enables information to become globally accessible for processes such as working memory, reporting, and behavioral control. In contrast, unconscious perceptual content is thought to occur within domain-specific perceptual systems, without involvement of higher-order (association) processing regions. Moreover, since the GNWT highlights a clear distinction between unconscious and conscious information processing in terms of non-linear dynamics in the neural response that occur in fronto-parietal cortex during conscious processing, the GNWT does not predict significant generalization between the brain representation of conscious and unconscious contents. Therefore the GNWT may need to be revised in order to account for our results.

Higher-order theories of consciousness also suggest that the prefrontal cortex is critical for consciousness. However, according to higher-order theories, the role of prefrontal cortex in consciousness relates to the re-representation of the content being represented in downstream sensory regions (i.e. meta-representation). Higher-order theories allow for a significant scope of unconscious processing in brain and behavioural responses, and they do not simply map consciousness with stronger prefrontal signals (Brown et al., 2019). The meta-representational role of the prefrontal cortex that is necessary for consciousness according to the higher-order view likely depends on long-range feedback connections (Huang et al., 2020) and also on other complex neural coding schemes and operations that are difficult to measure with fMRI. Additional work is needed to make further determinations.

## Data availability statement

Analysis scripts are available at https://github.com/nmningmei/unconfeats. The fMRI data can be found at https://openneuro.org/datasets/ds003927.

## Supplementary materials

**Figure 7:**
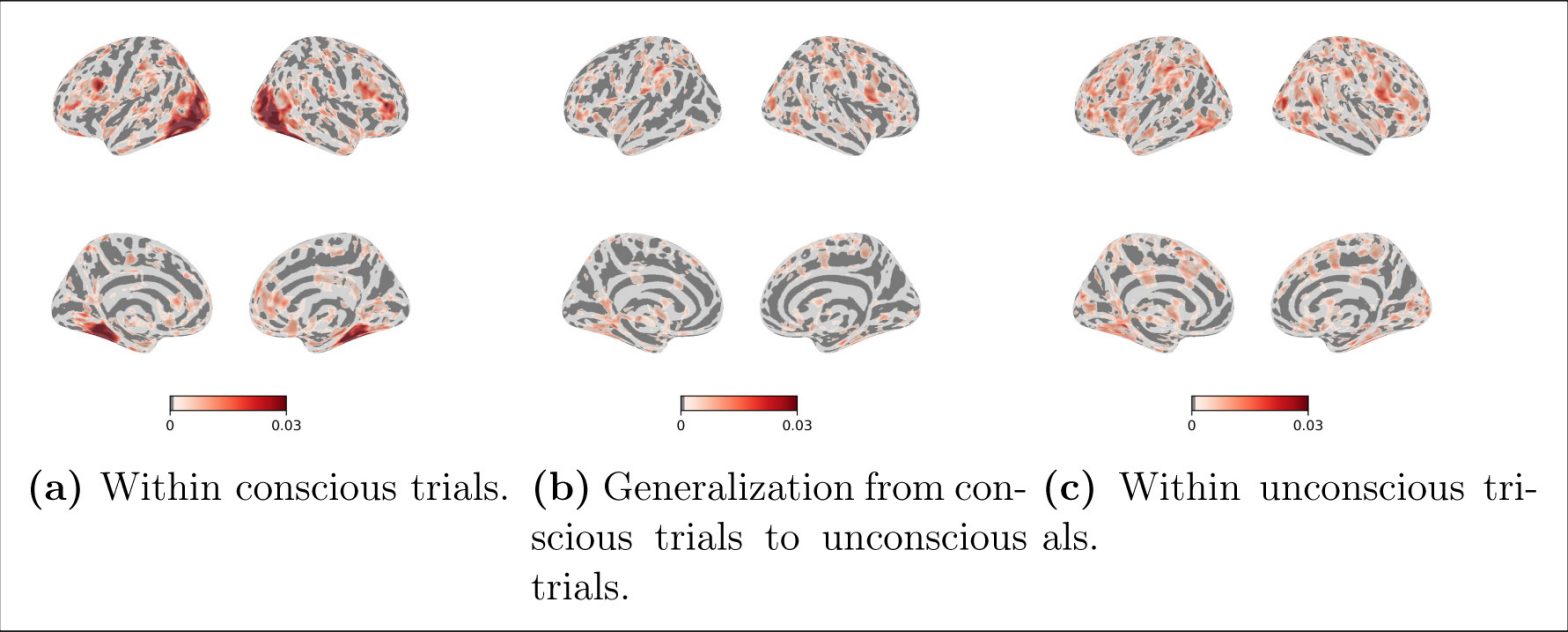
**Searchlight RSA results in different cross-validation procedures using VGG19 as the encoding model**. **(a)** within the conscious trials, **(b)** generalization from conscious to unconscious trials, and **(c)** within the unconscious trials. Correlation coefficients were assigned to the center of the moving searchlight sphere. We show significant clusters that have been whole-brain corrected using TFCE (*p* < 0.05). The color bars show the range of correlation coefficients between 0 and 0.03. The left/right images of each panel display the left/right hemisphere.
